# A Quality Initiative Reducing Adverse Outcomes in Pediatric Patients with DKA During Intrafacility Transit

**DOI:** 10.1097/pq9.0000000000000194

**Published:** 2019-07-22

**Authors:** Michael J. Stoner, Kelli S. Burkey, Daniel M. Cohen

**Affiliations:** From the * Department of Pediatrics, The Ohio State University College of Medicine, Columbus, Ohio; † Pediatric Emergency Department, Nationwide Children’s Hospital, Columbus, Ohio.

## Abstract

**Introduction:**

Treatment of diabetic ketoacidosis (DKA) requires close and timely monitoring to prevent serious adverse events. This quality improvement project details how our institution improved blood glucose monitoring around hospital admission. The project aimed to increase the blood glucose assessments for children with DKA receiving insulin in the emergency department (ED) within 30 minutes before transitioning to an inpatient unit.

**Methods:**

We implemented a series of Plan-Do-Survey-Act (PDSA) cycles established by a multidisciplinary team for this project, with the primary outcome of obtaining the blood glucose level within 30 minutes before leaving the ED and secondarily preventing episodes of hypoglycemia. These PDSAs harnessed the electronic health record, to prompt and direct the medical staff, to improve blood glucose monitoring.

**Results:**

From March 2015 to November 2017, we saw 640 patients in our ED for DKA. Of these, we admitted 629 to the inpatient unit with treatment that included continuous infusion of insulin. Over this period, we increased blood glucose monitoring for these patients within 30 minutes before the transition from 56% to >90%. Following the final PDSA cycle, we observed no reported episodes of hypoglycemia.

**Conclusion:**

Using the functionality of the electronic health record, we showed significant, rapid, and sustained increases in compliance with the International Society for Pediatric and Adolescent Diabetes guideline by alerting ED staff caring for patients receiving continuous insulin around the time of care-team transitions. We believe that this program is easily replicable, cost-effective, and safety enhancing.

## INTRODUCTION

The 2014 National Action Plan for Adverse Drug Event Prevention of the Office of Disease Prevention and Health Promotion (ODPHP) identified that diabetes agents were one of the 3 highest priority drug classes.^1^ Within this class, insulin specifically requires tight control and close monitoring. Further, the Institute for Safe Medication Practices (ISMP) also identifies insulin as a high alert medication, as does the pediatric-specific literature.^2–4^

Each year 5%–7% of children with type 1 diabetes develop diabetic ketoacidosis (DKA).^5–7^ Treatment for DKA involves replacing bodily fluids, insulin, and electrolytes to reverse the process and resume normal metabolic function. Close monitoring and tight glycemic control are of the utmost importance in treating children with DKA. In contrast to DKA treatment in adults, the International Society for Pediatric and Adolescent Diabetes (ISPAD) recommends a slower correction process over 48 hours with regimented reduction of serum glucose (50–150 mg/dl per hour) with hourly monitoring due to insulin’s ability to decrease blood glucose rapidly.^8–10^ A sudden drop in blood sugar can result in altered mental states, seizures, and coma, and has been associated with cerebral edema during DKA treatment.^11,12^ Adherence to a slower glucose correction and strict monitoring during DKA treatment is necessary to avoid complications such as hypoglycemia and cerebral edema, which are a major cause of death in children and adolescents presenting with DKA.^13,14^

Given that decreased monitoring increases the chance of complications, patient handoffs are frequent times of risk for medical errors.^15,16^ Our institution identified critical delays-in-care due to a lack of blood glucose monitoring for children with DKA receiving insulin in the interval between the emergency department (ED) admission decision and resuming care on the inpatient unit. Utilizing our event reporting system for 3 years before the beginning of this project, we identified 14 related adverse events including poor monitoring, rapid decline in blood sugar, worsening DKA, and episodes of hypoglycemia. A review of the current literature found no reports focused specifically on transitions of care during diabetes treatment; but one study identified lack of monitoring as a leading cause of medication error related adverse drug events (ADEs) in general.^17^

This quality improvement (QI) initiative focuses on improving care and safety for children in a state of DKA. Using standard QI methodology based on the Institute of Healthcare Improvement’s model, the specific aim was to increase the percentage of physician-reviewed blood glucose checks on children with DKA in the ED while receiving continuous infusions of insulin within the 30 minutes before hospital admission.^18^ This effort is the first of its kind in the pediatric literature.

## METHODS

The Institutional Review Board (IRB) chair waived the formal approval process for this QI project. This article follows the SQUIRE 2.0 guidelines for QI reporting excellence.^19^

### Context

Our hospital is an urban, freestanding, pediatric, tertiary care center. The 62-bed ED, which serves a catchment area encompassing 3 states, is a level 1 pediatric trauma center and sees approximately 90,000 children annually. The hospital’s electronic health record (EHR) is Epic Software (Epic Systems Corporation, Verona, Wis.).

We formed a multidisciplinary team, including nursing staff (ED and endocrine inpatient unit), ED paramedics, an ED pharmacist, clinical informatics specialists, and pediatric emergency medicine physicians. In compliance with ISPAD clinical practice consensus guideline recommendation of hourly testing, the team identified and prioritized consistent monitoring with notification to the treating physician of blood glucose levels (BGLs) within 30 minutes before departure from the ED. This 30-minute time was chosen to minimize adverse events associated with transitioning to the inpatient unit, thus allowing additional time for adjustments in the child’s care, transit time, and resumption of active care in the inpatient unit. This final BGL was done primarily by capillary sampling via bedside point of care testing.

### Patient Population

We evaluated the charts of children presenting in the ED with DKA between March 2015 and November 2017. Eligible patients included all children administered insulin via continuous intravenous drip and subsequently admitted to the hospital. We excluded patients who were treated by an alternative method, received insulin for another reason, or discharged to home.

### Interventions

Our first Plan-Do-Study-Act (PDSA) cycle (phase I) incorporated a best practice alert (BPA) presented to the physician’s screen in a child’s EHR. The BPA would prompt the physician to order the final ED BGL check and provide a link to the order. This BPA would display at the time of attending the ED or a fellow entered the order for the hospital admission. The BPA functionality was linked to the insulin order so that it would only display if the patient had a current order for continuous insulin (Table 1).

**Table 1.**
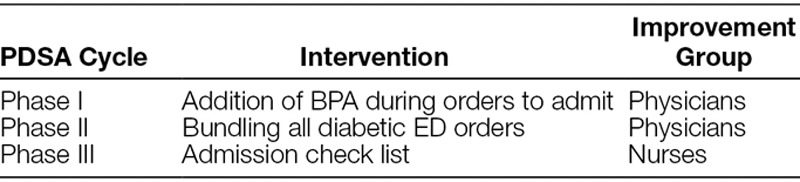
Plan-Do-Study-Act Phases and Areas of Intervention

In the second PDSA cycle (phase II), we initiated an order set into the EHR software. This order set bundled all orders necessary for the evaluation and treatment of children with diabetes, including those presenting with DKA. The order set included orders for fluids, insulin, monitoring parameters, laboratory tests, and a preadmission bedside BGL check within 30 minutes before departure from the ED. Additionally, individual orders were removed from the order preference list, thus constraining the physician to use the order set containing these bundled orders.

The final PDSA cycle (phase III) focused on a computerized reminder for the nurses caring for the patient. We placed an electronic “stop” in the EHR of every child admitted with a history of diabetes on their problem list or an insulin order placed in the ED. This “stop” was part of the ED nurse’s preadmission checklist and required the nurse to input the preadmission BGL and mark a box indicating that they informed the treating physician before patient departure from the ED. The omission of this part of the final admission process prohibited the electronic transfer of the child’s EHR from the ED to the inpatient track board. This additional “stop” addressed both needs: a reminder to test the BGL and to notify the child’s treating ED physician with results.

### Measures

The primary outcome of this project was obtaining the BGL within 30 minutes before leaving the ED. Secondary outcomes evaluated overall rates of hypoglycemia and/or development of cerebral edema. For balancing measures, we tracked ED time durations for these patients, due to potential increased time for task completion by nurses and medics and communication with physicians. We also tracked the overall ED length of stay for all medical patients admitted with the same triage acuity level.

## RESULTS

From March 2015 to November 2017, 640 children visited our pediatric ED with the diagnosis of DKA. Of those, we admitted 629 (98.3%) to the hospital receiving a continuous insulin infusion and included them in this QI initiative. Three PDSA cycles, using EHR functionality, allowed us to achieve >90% compliance as highlighted below.

### Primary Outcome

**Fig. 1.**
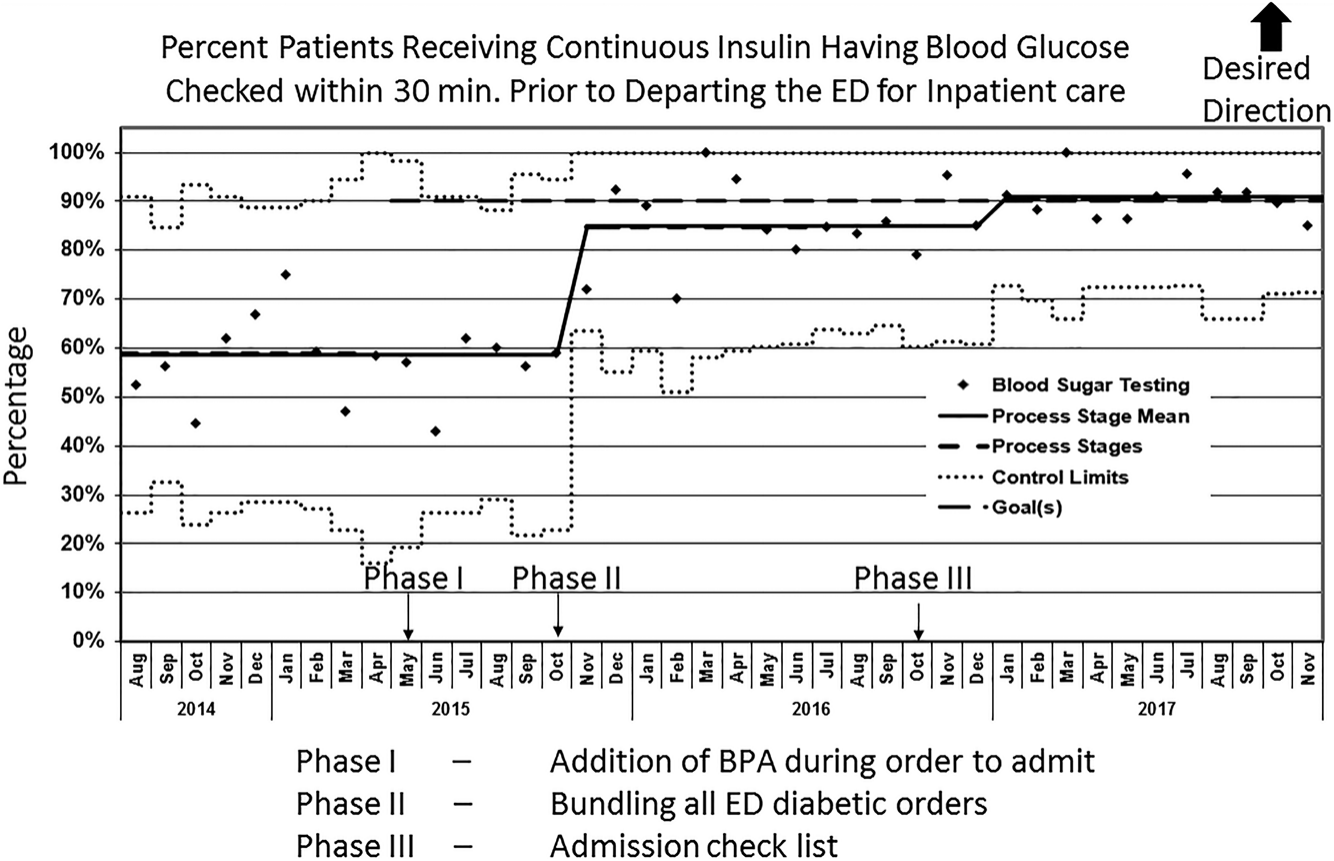
P-chart. Percent patients receiving continuous insulin having blood glucose checked within 30 minutes before departing the ED for inpatient care.

During this project, we achieved our aim of obtaining a BGL value within the 30-minute window before leaving the ED for >90% of included patients and sustained for >6 months. Phase I, incorporating a pop-up BPA within the EHR, instigated a slight increase toward our aim but no substantial change in our baseline (Fig. 1). However, phase II bundled all ED orders necessary for evaluation and treatment of diabetes, and showed the greatest improvement toward the specific aim with a rise from 56% (baseline) to 85% (Fig. 1). Our final PDSA cycle (phase III), which added the electronic “stop” to the final nursing preadmission checklist, exhibited the greatest improvements with compliance in obtaining a BGL value within the 30-minute window before leaving the ED. This improvement exceeded our 90% compliance goal within 20 months of the first PDSA cycle, and we sustained the improvement for the final year of the project (Fig. 1). From start of the project until going into sustain mode, (March 2015 to November 2017), there were 41 individual patient visits for DKA (8%), among those with improved monitoring of blood sugar within 30 minutes of departing the ED, which resulted in a change in management.

### Secondary Outcomes

**Fig. 2.**
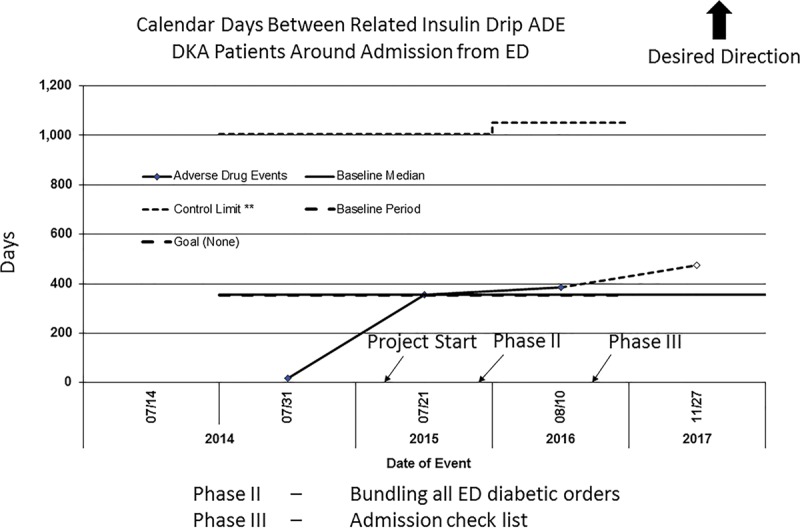
G-Chart. Calendar days between related insulin drip ADE DKA patients around admission from ED.

Utilizing our event reporting system for 3 years before the beginning of this project, we identified 14 related adverse events, including poor monitoring, rapid decline in blood sugar, worsening DKA, and episodes of hypoglycemia. While not a large number, this still relates to 1 event every 2–3 months. We found no adverse events reported into the hospital’s ADE system (including hypoglycemia episodes or urgent order for adjustment in glucose-containing fluids secondary to overly rapid drops in blood sugar) associated with ED glucose monitoring since implementing phase III of the QI project (Fig. 2). Further, we had no reported episodes of cerebral edema requiring IV mannitol due to lack of monitoring.

### Balancing Measures

**Fig. 3.**
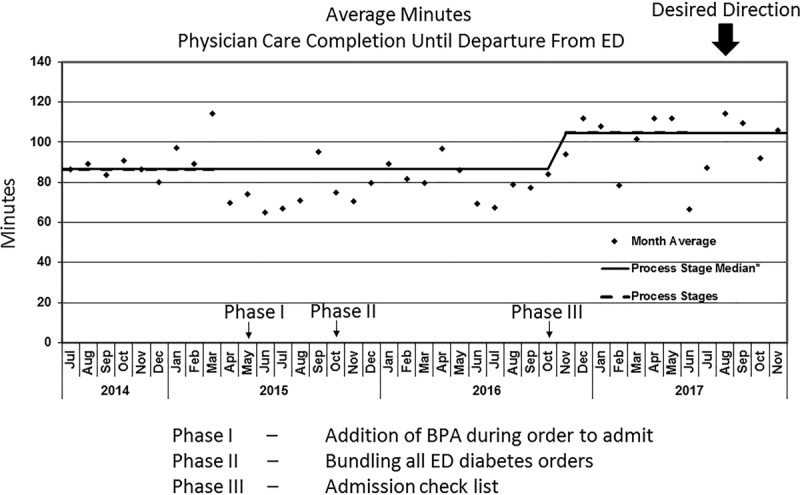
P-Chart balancing measure. Average minutes: physician care completion until departure From ED.

**Fig. 4.**
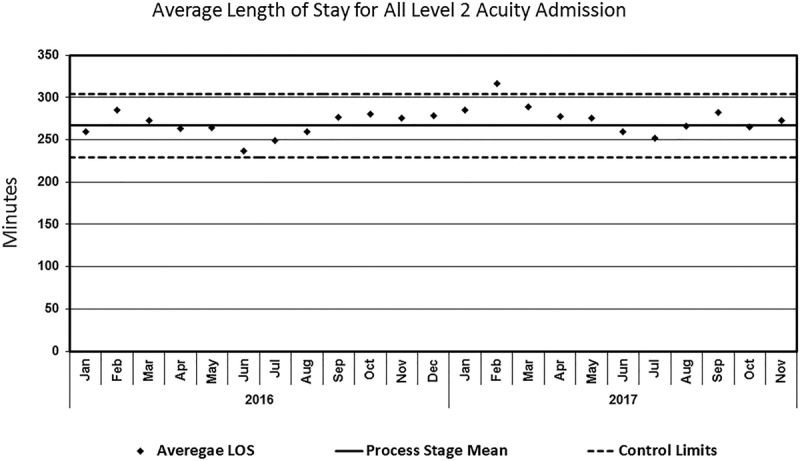
Balancing measure: run chart. Average length of stay of all level 2 acuity admission. LOS, length of stay.

The time between completion of care by the providing doctor until the patient departure from the ED (Fig. 3) shows a shift in the baseline from 87 to 114 minutes. This finding indicates the development of a 27-minute increase in care time when we implemented our final PDSA, phase III. Patients in the ED are triaged using an emergency severity index (ESI) scaled from 1 to 5 with 1 being the most emergent and 5 being the least.^20,21^ Typically a patient with an ESI score of 1 should be seen immediately, and a patient with an ESI score of 2 should optimally be seen within 10–20 minutes. As we classify these DKA patients as an ESI category 2, we also looked at the time between completion of care by the providing doctor and the patient departure from the ED for all admitted ESI category 2 patients (Fig. 4). For this measure, we did not find a shift in the baseline, suggesting this QI intervention did not affect the overall flow for admitted ESI category 2 patients in the ED. To expand on this further, we looked at all patients in the ED for significant changes in the length of stay and did not find any.

## DISCUSSION

This QI initiative successfully achieved our aim of testing 90% of patients who were receiving continuous insulin with a BGL check within 30 minutes before departing the ED for intrahospital transfer. Using the functionality within EHR, we improved our rate from 56% to over 90% and sustained compliance for >6 months.

In the treatment of DKA, ISPAD recommends a gradual correction of serum glucose (50–150 mg/dl per hour) with monitoring every 60 minutes.^8–10^ This regimen mitigates the high risk of continuous insulin resulting in hypoglycemia, which can lead to altered mental states, seizures, coma, prolonged length of stay, and possibly cerebral edema.^11,12^ A lack of monitoring is a chief cause of medication-related ADEs.^15^ Given our institutional history for lapses in blood glucose monitoring, especially in the context of known periods of susceptibility to errors during transition and handoffs, we chose to work on this vulnerable period.^22,23^

In this study, we highlight 3 PDSA cycles related to orders in the EHR. We intentionally escalated these interventions in intensity, from presenting an order reminder, to nudging use of order sets and finally to force the use of the order by using an EMR “hard stop” preventing admission without order completion. Although there was an incremental change, phase I was least effective. We speculate that this was due to provider misinterpretation of the BPA, alert fatigue, and QI project completion. Prior studies have described such phenomena and the issues with excessive pop-ups causing information overload, delays, and even medical errors.^24–26^ This motivated us to remove this BPA and move on to phase 2, which yielded a significant change. We believe this was due to gently forcing providers to utilize order sets and having the BGL order “prechecked.” However, because the BGL order was categorized as pro re nata and not time-delimited, nurses could opt not to perform the final BGL monitoring order. Additionally, for this phase, there was no requirement to document communication around the BGL result.

In our final phase, we added a mechanism to increase communication of critical results, one of the 2018 Joint Commission Hospital National Patient Safety Goals.^27^ Given the often fast-paced, high stakes, multidisciplinary environment of care in the ED and frequent handoffs and transfers, enhancing communication is highly important. In such a setting, practitioners of varying disciplines, experience, and educational levels may be task switching and easily distracted. Left unchecked this situation is a setup for medical errors. Logically, a “time out” prompt or checklist within this heterogeneous environment is needed. The surgical and airline literature suggest that a checklist works to decrease errors and increase teamwork and communication.^28–30^

Consequently, when we added a checklist prompt in the final phase, we achieved >90% compliance of our project’s goal. This increased BGL compliance that was sustained for 6 months and, importantly, since reaching our goal, there were *no* insulin-related ADEs associated with transition-of-care from our pediatric hospital ED to the inpatient care unit. Of note, throughout the project, approximately 8% of the admitted patients in DKA making the 30-minute cutoff had a change in their management due to this intervention that, if left unchecked, could have resulted in an ADE. This change was mostly a result of blood glucose reaching a level where the glucose-containing fluid infusions needed to be increased to prevent hypoglycemia.

We explored ED length of stay as a balancing measure and found that increased compliance with BGL monitoring was associated with an increase (27 minutes average) in the patient’s admitting process. Although the increased length of stay of our DKA patient is often perceived negatively, this time may identify and manage critical interventions before care transition. Our secondary balancing measure, the length of stay for all admitted patients with a similar acuity, did not show a significant change, indicating that our initiative only affected the intended patients.

### Limitations

As a single-center study, these findings may not be generalizable to other settings. Our institution is a large, tertiary care pediatric academic center, with an average of 20 children per month requiring admission for DKA. Our institution has a robust QI culture with a dedicated support system. Additionally, EDs often have multiple, simultaneous initiatives, which can be potentially synergistic and/or distracting. Finally, we have strong informatics support and participation in our multidisciplinary team. We chose to use EHR tools, such as “pop-up” reminders and hard stops, which individual institutions may choose to deploy sparingly. Thus, applicability to different organizations may require alternate adaptations.

## CONCLUSIONS

For the treatment of DKA with continuous intravenous insulin, embedding a set of QI interventions into the EHR introduced significant, rapid, and sustained increases in compliance to the International Society for Pediatric and Adolescent Diabetes guidelines for monitoring BGLs and improved patient safety. This program is easily implementable, and we suggest that other organizations use this relatively simple technique to improve their results if they are struggling to achieve high levels of ISPAD compliance and reductions in insulin ADEs.

## ACKNOWLEDGMENTS

We recognize the following people for their efforts in this project: Don Buckingham, MBOE, for his efforts with data collection and analysis, and Melody L. Davis, PhD, for her assistance with manuscript preparation. We also thank the residents, fellows, and emergency nurses for their assistance in making this project successful.

## DISCLOSURE

The authors have no financial interest to declare in relation to the content of this article.
